# Promoting healthful family meals to prevent obesity: HOME Plus, a randomized controlled trial

**DOI:** 10.1186/s12966-015-0320-3

**Published:** 2015-12-15

**Authors:** Jayne A. Fulkerson, Sarah Friend, Colleen Flattum, Melissa Horning, Michelle Draxten, Dianne Neumark-Sztainer, Olga Gurvich, Mary Story, Ann Garwick, Martha Y. Kubik

**Affiliations:** School of Nursing, University of Minnesota, 5-160 Weaver-Densford Hall, 308 Harvard Street SE, Minneapolis, MN 55455 USA; Department of Family Medicine & Community Health, University of Minnesota, Minneapolis, Minnesota USA; Division of Epidemiology & Community Health, University of Minnesota, Minneapolis, Minnesota USA; Community & Family Medicine and Global Health, Duke University, Durham, North Carolina USA

**Keywords:** family based interventions, childhood obesity, clinical trials, behavioral strategies, prevention, family meals

## Abstract

**Background:**

Family meal frequency has been shown to be strongly associated with better dietary intake; however, associations with weight status have been mixed. Family meals-focused randomized controlled trials with weight outcomes have not been previously conducted. Therefore, this study purpose was to describe weight-related outcomes of the HOME Plus study, the first family meals-focused randomized controlled trial to prevent excess weight gain among youth.

**Methods:**

Families (*n* = 160 8-12-year-old children and their parents/guardians) were randomized to intervention (*n* = 81) or control (*n* = 79) groups. Data were collected at baseline (2011–2012), post-intervention (12-months post-baseline) and follow-up (21-months post-baseline). The intervention included ten monthly group sessions (nutrition education; hands-on meal and snack planning, preparation, and skill development; screen time reductions) and five motivational, goal-setting phone calls. The main outcome was child body mass index (BMI) z-score.

**Results:**

General linear models, adjusted for baseline values and demographics, showed no significant treatment group differences in BMI z-scores at post-intervention or follow-up; however, a promising reduction in excess weight gain was observed. Post-hoc stratification by pubertal onset indicated prepubescent children in the intervention group had significantly lower BMI z-scores than their control group counterparts.

**Conclusions:**

The study used a strong theoretical framework, rigorous design, quality measurement and a program with high fidelity to test a family meals-focused obesity prevention intervention. It showed a modest decrease in excess weight gain. The significant intervention effect among prepubescent children suggests the intervention may be more efficacious among relatively young children, although more research with appropriately powered samples are needed to replicate this finding.

**Trial registration:**

This study is registered at www.clinicaltrials.gov NCT01538615. Registered 01/17/2012.

## Background

Overweight/obesity continues to be major public health problem for youth, with prevalence among 2-19-year-olds at about one-third in the United States [1] and slightly lower but increasing rates worldwide [2]. To address this issue, both childhood obesity prevention and treatment randomized controlled trials (RCTs) have been initiated. Historically, childhood obesity *prevention* trials have consisted of initiatives with children of all sizes with implementation in school [3] and community settings [4, 5] while *treatment* trials have typically focused on overweight youth within clinics and primary care facilities [6, 7]. Prevention-focused RCTs have been mildly to moderately successful in reducing body mass index (BMI) [3, 8], particularly among children compared to adolescents [8, 9]. Treatment studies with overweight youth typically have larger effects on BMI [10] but are expensive [11]. Prevention of excess weight gain *and* the reduction of excess weight are critical for the future of our youth.

Perhaps interventions would be most effective if they used successful approaches from both types of programs. Successful prevention and treatment programs with significant effects on BMI are more likely high-quality studies with solid theoretical foundations, rigorous design, quality measurement and clear descriptions of intervention delivery integrity, fidelity and dose [3, 12, 13]. Additionally, successful programs often include family involvement, behavioral monitoring, environmental changes and longer-term interventions [8, 10, 14]. National experts have recommended that programs improve the family’s ability to support children’s weight-related behavior change [11]; however, including parents in childhood obesity trials can be challenging [10]. Family-based approaches for childhood obesity prevention interventions have increased over the past several decades and those rooted in behavior theory appear to have more successful outcomes for overweight youth [15]. Historically, treatment programs have been more likely than prevention programs to include parents and focus on the home environment and often teach about self-monitoring, goal setting, problem-solving and have explicit behavioral change expectations [7]. More recently, childhood obesity prevention RCTs have emphasized the importance of the home environment and family involvement in reducing childhood obesity and have included adiposity-related outcomes [16]. However, few have been conducted with school-age or preadolescent children even though these children are at high risk of excess weight gain and obesity [17]. Moreover, few studies that measure potential effects over a longer post-intervention period have been conducted to evaluate sustainability [9].

One growing area of research that may be promising for obesity prevention is family meals promotion. Noninterventional, cross-sectional and longitudinal studies have demonstrated robust findings between family meal frequency and child dietary intake quality [18–21], with some studies showing promising associations between family meal frequency and weight, particularly among young children [21, 22]. However, few intervention programs have specifically examined promoting healthful and frequent family meals as a method of addressing childhood obesity. Our study team conducted a pilot RCT with a family meals-focused program in which nutrition and weight-related outcomes were directly measured; findings were promising but, as a pilot, it was not powered to assess change in weight-related outcomes [23, 24]. A few other programs reported in the literature promoted family meals but not as the primary focus [16, 25, 26] or did not evaluate weight-related outcomes [27, 28]. The present RCT builds upon the extant literature as it was designed to evaluate the efficacy of a family meals-focused intervention to prevent excess weight gain in 8-12-year-old children. It incorporated both childhood obesity prevention and treatment approaches. The a priori hypothesis was that children whose families participated in the intervention program would have significantly lower BMI z-scores at post-intervention (12 months post-baseline) compared to children in the control group; similar findings were hypothesized to be sustained at follow-up (21 months post-baseline). Given the strong associations between puberty and weight gain in the literature [29–31] and robust findings that younger children eat meals more often with their families than older children and may thus be more influenced by changes in family meals [20, 32], a subsequent aim was added to examine whether the intervention had a differential effect by pubertal onset.

## Methods and procedures

### Trial design

The Healthy Home Offerings via the Mealtime Environment (HOME) Plus study was designed as a two-group RCT (intervention and control) to promote healthier eating, reduce sedentary behaviors (i.e., meal-related screen time) and prevent excess weight gain among 8-12-year-olds by increasing the frequency and healthfulness of family meals [33]. A staggered-cohort design was used in which two cohorts of families from a large metropolitan area in the upper US Midwest were recruited and randomized to treatment groups one year apart (2011 and 2012). Fully detailed power calculations are published elsewhere [33]. We partnered with the Minneapolis Park and Recreation Board because their mission promotes health, well-being and community and they have a significant presence in the local area. Six Minneapolis Park and Recreation community centers were selected as intervention sites based on the economic- and cultural-diversity of the populations they serve and site logistics (e.g., kitchen facilities).

### Participants

Staff and volunteers recruited 160 children and one of their parents/guardians (the primary meal-preparing adult in the household) from community centers using flyers, targeted email lists, and in-person presentations/discussions; some participants learned of the study by word of mouth. Child inclusion criteria were as follows: being 8–12 years old, having an age- and gender-adjusted BMI percentile above the 50^th^ percentile (to potentially target a more at-risk group), having English literacy, and living with the participating parent/guardian most of the time. Exclusion criteria (children and parents) were plans to move from the area within 6 months, and medical conditions prohibiting participation (e.g., extreme food allergies). Regarding program reach, the HOME Plus sample appears to be similar in racial/ethnic diversity and slightly more educated compared to residents in the county where the community centers were located [34].

### Procedures

Participating parents and children signed consent and assent forms, respectively. Data were collected by research staff at baseline, post-intervention (12-months post-baseline to assess intervention efficacy) and follow-up (21-months post-baseline to assess sustainability). Baseline data collection occurred almost exclusively at participants’ homes (99 %). At post-intervention and follow-up, 35 %-45 % of families selected community sites for data collection. Families received a gift card at each data collection visit. All study procedures and materials were approved by the University of Minnesota IRB.

### Randomization

**Fig. 1:**
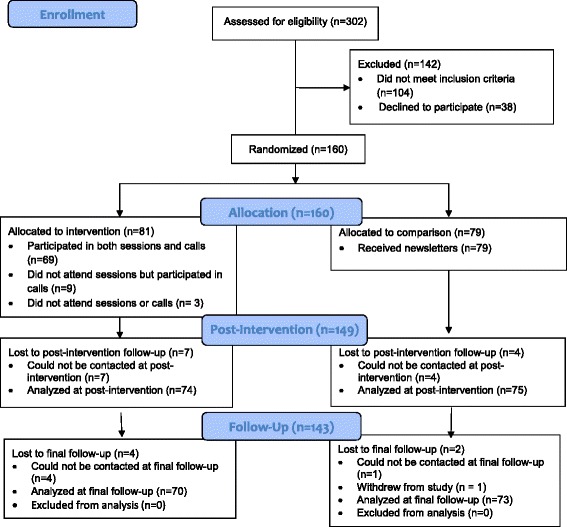
Consort diagram for HOME Plus study

After baseline assessments, the study statistician randomized families to the HOME Plus intervention (*n* = 81) or control (*n* = 79) groups within each community center using a computer-generated randomization schedule (nQuery Advisor version 6.01, Statistical Solutions, Ltd.). Assignment was not blinded. Fig. 1 depicts a consort flow diagram for sample sizes at recruitment, randomization, data collection and analysis. Additional details of the study design and protocols are published elsewhere [33, 35].

### Measures

Children and parents completed psychosocial surveys, and trained staff measured height and weight using standardized procedures [36]. Parent surveys included demographic information. Family dinner frequency was assessed with the following question: During the past seven days, how many times were you sitting *and eating* with your child when he/she ate his/her *dinner*? Response options included 0 to 7 times. Children completed a validated assessment of their pubertal maturation using the Pubertal Development Scale which assessed for skin changes, growth spurts, body hair, voice changes (boys only), and breast development and menarche (girls only) [37]. Height and weight were used to calculate BMI values that were standardized for age and gender using CDC Guidelines [38]. Child weight status was classified as follows: BMI < 85^th ^% = normal weight, 85^th^ ≤ BMI < 95^th ^% = overweight, and BMI ≥ 95^th ^% = obese. The primary study outcome was child BMI z-score at post-intervention.

### Intervention

Social Cognitive Theory [39] and a socio-ecological framework [40] guided measurement of key variables and the development of the HOME Plus intervention to address personal, behavioral and environmental factors associated with healthful home food environments; sedentary behavior, including screen time; meal and snack times, including preparation; and food and beverage consumption within family homes. The intervention was delivered by trained staff (registered dietitians and a public health nurse) and targeted family change in the planning, frequency and healthfulness of family meals and snacks and limiting meal-related screen time. The intervention included ten monthly group sessions (see Table 1) and five brief goal-setting telephone calls [35]. Families received a guidebook with session topics, strategies to promote behavior change and study goals, recipes and community resources. All family members were invited to attend sessions and transportation and childcare were provided, if needed. The most commonly used behavior change taxonomy clusters used in the sessions included *Goals and Planning, Social Support, Repetition and Substitution, Natural Consequences, Shape Knowledge, and Antecedents* [41] with many associated behavior change techniques as shown in Table 1. The goal-setting calls (~20 minutes) were completed by dietitians trained in motivational interviewing who tailored each call to the family-selected behavioral goal(s). Calls included the same behavior change techniques as in-person sessions but followed an interview format, utilized motivational interviewing techniques [42] and provided opportunities to discuss behaviors/goals that complemented the group session topics. Control group participants received a monthly family-focused newsletter and did not receive the HOME Plus intervention program.

**Table 1:** HOME Plus study intervention content by session with behavioral change clusters and techniques [41] used

Session Name	Topics	Behavior Change Technique Clusters Used	Behavior Change Technique Cluster Components Implemented
Within all sessions	• Check-in/opening discussion	Feedback and monitoring	Others monitoring with awareness Feedback on behavior Self-monitoring of behavior Self-monitoring of outcome of behavior
		Goals and planning	Goal setting (behavior) Problem solving/coping planning Goal setting (outcome) Action planning (including implementation of intentions) Review behavior goal(s) Discrepancy between current behavior and goal standard Review outcome goal(s) Commitment
		Social support	Social support (general)
	• Taster’s Choice (Taste-testing)	Associations	Exposure
		Repetition and Substitution	Behavioral rehearsal/practice Habit formation
		Comparison of Behavior	Modeling of behavior
		Social support	Social support (general)
	• Taster’s Choice (Taste-Testing) Homework	Reward and Threat	Incentive
	• Cooking	Shaping knowledge	Instruction on how to perform a behavior
		Repetition and Substitution	Behavioral rehearsal/practice Habit formation
	• Family Meal	Social Support	Social support (general)
		Comparison of Behavior	Modeling of behavior
		Shaping knowledge	Instruction on how to perform a behavior
		Repetition and Substitution	Behavioral rehearsal/practice
1-Let’s get started	• Best family meal ever • Wash, chop, slice and safety…kitchen basics	Natural Consequences	Health consequences Self-assessment of affective consequences
		Goals and planning	Goal setting (behavior) Problem solving/coping planning Goal setting (outcome) Action planning (including implementation of intentions) Review behavior goal(s) Discrepancy between current behavior and goal standard Review outcome goal(s) Commitment
		Social Support	Social support (general)
		Shaping knowledge	Instruction on how to perform a behavior
		Repetition and Substitution	Behavioral rehearsal/practice
2-Ready, Set, Goal	• Goal setting…breaking it into bite-size pieces • Let’s give them something to talk about–conversation starters • Recipe revolution–common abbreviations	Goals and planning	Goal setting (behavior) Problem solving/coping planning Goal setting (outcome) Action planning (including implementation of intentions) Review behavior goal(s) Discrepancy between current behavior and goal standard Review outcome goal(s) Commitment
		Shaping knowledge	Instruction on how to perform a behavior
		Repetition and Substitution	Behavioral rehearsal/practice
3-Thinking outside the box	• Switch it up: meal planning makeovers • Go! Slow! Whoa! • Successful recipes = accurate measures	Antecedents	Restructuring the physical environment
		Repetition and Substitution	Behavioral rehearsal/practice Behavior substitution
		Social Support	Social support (general)
		Shaping knowledge	Instruction on how to perform a behavior
4- What’s for dinner 2night?	• Cook today, eat tomorrow–or freeze for another day • READ it before you EAT it • A dash of this, a pinch of that–measuring ingredients	Social Support	Social support (general)
		Comparison of outcomes	Pros and cons
		Repetition and Substitution	Behavioral rehearsal/practice Habit formation
		Shaping knowledge	Instruction on how to perform a behavior
5-Too much? Not enough?	• Portion distortion– helpings, portions and servings • Are you hungry? Full? Listening to your body’s cues • Get creative-colorful, fresh and nutritious salads	Feedback and Monitoring	Self-monitoring of behavior Self-monitoring of outcome of behavior
		Repetition and Substitution	Habit formation
		Shaping knowledge	Instruction on how to perform a behavior
6-Keep it under wraps	• Fast, fun and full of acceptance–ideas for picky eaters • Making sense of advertising • Wrap it up-Quick and easy meals	Shaping knowledge	Instruction on how to perform a behavior
		Comparison of Behavior	Modeling of behavior
		Natural consequences	Self-assessment of affective consequences
7-Balance, balance, keep the balance	• Healthy snacks-beyond apples and oranges • The race is on…choosing healthy snacks • Peel! Chop! Fruits!	Repetition and Substitution	Behavioral rehearsal/practice Behavior substitution Habit formation Habit reversal
		Natural Consequences	Health Consequences
		Antecedents	Restructuring the physical environment
		Associations	Prompts/cues
		Shaping knowledge	Instruction on how to perform a behavior
8-Less sugar and fat–a sweet deal	• Sip smarter–the bottom line on sugary drinks • Which snack or beverage? Check the facts! • Peel! Chop! Vegetables!	Repetition and Substitution	Behavioral rehearsal/practice Behavior substitution Habit formation Habit reversal
		Natural Consequences	Health Consequences
		Antecedents	Restructuring the physical environment
		Associations	Prompts/cues
		Shaping knowledge	Instruction on how to perform a behavior
9-AGREENable meals and snacks	• Why your choices matter • Celebrate seasons–picking produce that’s fresh & less expensive	Repetition and Substitution	Behavioral rehearsal/practice Behavior substitution Habit formation Habit reversal
		Natural Consequences	Health Consequences
		Antecedents	Restructuring the physical environment
		Associations	Prompts/cues
		Shaping knowledge	Instruction on how to perform a behavior
		Comparison of Behavior	Modeling of behavior
10-The future is bright…planning ahead	• The Celebrity Chef is…you! • Kids can do it…families can do it	Repetition and Substitution	Habit formation
		Self-belief	Verbal persuasion to boost self-efficacy Focus on past success
		Identity	Self-affirmation Identity associated with behavior change
		Goals and planning	Goal setting (behavior) Problem solving/coping planning Goal setting (outcome) Action planning (including implementation of intentions) Review behavior goal(s) Discrepancy between current behavior and goal standard Review outcome goal(s) Commitment

### Program fidelity

To monitor and enhance program fidelity, observations of session delivery by the interventionists were conducted according to key established criteria [12, 43, 44] at sessions 3, 6, and 9 by trained university-level students using a standardized checklist. Analysis indicated 90 % of sessions were delivered as intended; the main protocol deviation was program start time as delays occurred when families did not arrive as scheduled.

### Dosage

Intervention “dose” was defined as the total number of in-person intervention sessions attended out of ten.

### Data analysis

Baseline comparisons between intervention and control groups were performed to examine any realized imbalance. Inclusion of covariates in the analytic models was determined by examining differences related to retention, correlations with BMI z-score and overlap between child and parent measures. Slight attrition occurred at post-intervention (see Fig. 1), with significantly lower retention among nonwhite participants and those receiving economic assistance. Child age was related to BMI, and child and parent race were significantly correlated with each other. Thus, general linear models to assess intervention effects on BMI z-score at post-intervention and follow-up were adjusted for baseline child BMI z-score, age, gender and race and family receipt of economic assistance. To examine the longitudinal intervention effect, a general linear mixed model was fitted, with baseline BMI z-score, treatment group, time (post-intervention and follow-up) and treatment group-by-time interaction as fixed covariates, adjusting for demographic covariates and including participant-specific random intercepts. We also explicitly modeled the partially-clustered nature of this sample (since the intervention was delivered in group settings with multiple families while control participants stayed “unclustered”) using a random coefficient multilevel model [45]. The intervention effect was not affected after adjustment for partial clustering and no intervention sub-group facilitation was observed; thus, only the results of the analyses conducted without the adjustment are presented. We assessed “dose–response” of the implemented intervention among intervention group participants who provided both baseline and post-intervention child BMI z-scores using multiple regression without and with adjustment for child age, gender, race and family economic assistance receipt at baseline. For post-hoc stratification to examine whether the intervention had a differential effect by pubertal onset, development scores were split at the median (development score: <1.6, *n* = 57, prepubescent vs. ≥1.6, *n* = 90, pubescent). This cut-point essentially categorized youth into prepubertal versus early puberty or beyond based on the original scale development [37] that is in line with Tanner Stages of prepuberty [31] (see Table 2). This variable was included in an interaction with treatment group in the regression models while controlling for demographic covariates. We were unable to use established puberty criteria such as menses for our cut-point as our 8–12 year old sample was relatively physically immature, as expected. Given the high frequency of family dinners at each assessment point (*M* = 4.7, SD = 2.0; *M* = 5.0, SD = 1.8; *M* = 5.1, SD = 1.7 at baseline, post-intervention and follow-up, respectively), for analysis, we modeled “frequent” (5 or more per week) family dinners using generalized linear mixed models (GLMM) with participant-specific random intercepts while controlling for baseline sociodemographic characteristics. Data management and statistical analyses were conducted using SAS software versions 9.2 and 9.3 (SAS Institute Inc., Cary, NC, USA). An intent-to-treat approach was used for all analyses. A two-sided type I error rate of 5 % was used to determine statistical significance.

**Table 2:** Baseline demographic characteristics of HOME Plus participants included in post-intervention analysis and by condition

Characteristics	Total analytic sample (*n* = 149 families)^a^	Intervention (*n* = 74 families)^a^	Control (*n* = 75 families)^a^	*p*-value
Child Demographics				
Age (M, SD)	10.3 (1.4)	10.5 (1.4)	10.2 (1.4)	.16
BMI z-score (M, SD)	.99 (.76)	.95 (.78)	1.02 (.74)	.59
Weight status				
BMI% < 85^th^	84 (56 %)	44 (60 %)	40 (53 %)	.75
85^th^% ≤ BMI% <95^th^	33 (22 %)	15 (20 %)	18 (24 %)	
≥95^th^	32 (22 %)	15 (20 %)	17 (23 %)	
Pubertal Development^b^				
Prepubescent	57 (39 %)	24 (32 %)	33 (45 %)	.11
Pubescent	90 (61 %)	50 (68 %)	40 (55 %)	
Gender (% female)	71 (48 %)	36 (51 %)	35 (49 %)	.81
Ethnicity/Race				
Hispanic				
White	106(71 %)	53 (72 %)	53 (71 %)	.75
Black	23 (16 %)	10 (13 %)	13 (17 %)	
Any other	20 (13 %)	11 (15 %)	9 (12 %)	
Parent Demographics				
Age (M, SD)	41.6 (7.6)	41.7 (7.9)	41.6 (7.3)	.94
BMI^c^ (M, SD)	28.3 (7.2)	27.1 (6.6)	29.5 (7.5)	.04
Weight status^c^				
Normal (BMI < 25)	59 (40 %)	32 (44 %)	27 (36 %)	.30
Overweight/Obese (BMI ≥ 25)	88 (60 %)	40 (56 %)	48 (64 %)	
Female gender	141 (95 %)	69 (93 %)	72 (96 %)	.45
Ethnicity/Race				
Hispanic				
White	120 (80 %)	60 (81 %)	60 (80 %)	.98
Black	19 (13 %)	9 (12 %)	10 (13 %)	
Any other	10 (7 %)	5 (7 %)	5 (7 %)	
Education				
≤High school	12 (8 %)	7 (10 %)	5 (7 %)	.30
Some college	45 (31 %)	18 (25 %)	27 (36 %)	
≥Bachelor’s degree	89 (61 %)	47 (65 %)	42 (57 %)	
Household Demographics				
Economic assistance (% yes)	52 (35 %)	30 (41 %)	22 (29 %)	.15

^a^Numbers may be reduced by varying small amounts because of incidental missing data. All statistics are n (%) unless noted otherwise.

^b^Pubertal Development is based on child self-reported scale (range 1–4). Prepubescent is a score of less than 1.6 and pubescent is greater than or equal to 1.6.

^c^Two pregnant women in the intervention condition were not included in BMI or weight status descriptives.

## Results

### Recruitment and retention

One-hundred-sixty families were recruited and randomized. There were no significant baseline weight-related or demographic (i.e., gender, race, age, education, economic assistance, cohort, site) differences between the intervention and control groups. There was high study retention at post-intervention and follow-up (see Fig. 1). Moreover, intervention participation was high with 85 % of families attending at least half of the in-person sessions and at least three of five motivational calls. Average attendance was 68 % for in-person sessions and 87 % for goal-setting calls over the ten-month intervention [35]. No serious adverse events were reported.

### Sample and baseline descriptives

Descriptive summaries of sample baseline measures are presented in Table 2. Average child age was about 10 years, slightly less than half were overweight/obese, and the majority was white. Parents’ mean age was about 41 years, and 60 % were overweight/obese. The majority was white and 95 % were women. Sixty-one percent of parent participants had at least a four-year college degree; 35 % received economic assistance through free/reduced lunch for their children or other public assistance.

### Intervention effects on BMI z-scores at post-intervention and at follow-up

Adjusted mean differences in BMI z-scores between control and intervention groups at post-intervention and at follow-up were not statistically significant and were estimated to be 0.03 (SE = 0.04; 95 % CI: −0.05, 0.12; p = 0.43) and 0.07 (SE = 0.05; 95%CI: −0.04, 0.17; p = 0.21), respectively (see Table 3). The only significant covariate was baseline BMI z-score with higher baseline scores associated with higher scores later.

**Table 3:** Multiple regression models for post-intervention and follow-up BMI z-scores

	Post-Intervention^a^ *n* = 149		Follow-Up^b^ *n* = 143	
Variable	β(SE)	*p*-value	β(SE)	p-value
Intercept	0.001 (0.19)	0.99	0.11 (0.22)	0.60
Group (C vs. I)	0.03 (0.04)	0.43	0.07 (0.05)	0.21
Baseline BMI z-score	1.00 (0.03)	<0.001	0.96 (0.04)	<0.001
Child gender (male vs. female)	0.01 (0.04)	0.80	−0.06 (0.05)	0.26
Child age (months)	−0.001 (0.001)	0.65	−0.00003 (0.002)	0.98
Child race				
White vs. Other	−0.03 (0.07)	0.63	−0.09 (0.08)	0.31
Black vs. Other	0.08 (0.08)	0.32	−0.06 (0.10)	0.53
Family economic assistance status (No vs. Yes)	0.03 (0.06)	0.57	−0.01 (0.07)	0.85

^a^12-months post-baseline; R^2^ = 0.90, *p*< 0.001

^b^21-months post-baseline; R^2^ = 0.86, *p*< 0.001

### Examples of the HOME Plus intervention effect on decreases in excess weight gain for an average non-overweight child and overweight child

Although not statistically significant, to facilitate understanding of the potentially clinically significant weight change associated with the HOME Plus intervention, we estimated corresponding decreases in expected weight gain associated *with our intervention effect* for an average 9- and 11-year-old at the 75^th^ and 85^th^ percentiles for BMI (corresponding to non-overweight and overweight) and 50^th^ percentile for height (all baseline) using the CDC growth charts [38]. For a 9-year-old at the 75^th^ percentile BMI, a corresponding decrease in expected average weight gain at post-intervention was about 0.18 kg; the decrease was more pronounced (around 0.22 kg) for a 9-year-old starting at the 85^th^ percentile (overweight) and corresponds to a post-intervention 84^th^ percentile (normal weight). Similarly, for an 11-year-old, the expected decreases in average weight gain at post-intervention were about 0.24 kg and 0.30 kg (at 75^th^ and 85^th^ percentiles, respectively).

### Longitudinal effect of the intervention on BMI z-scores

No statistically significant differences in BMI z-scores between treatment groups, within treatment groups over time or between treatment groups over time were observed when post-intervention and follow-up time points were modeled while adjusting for the baseline outcome values and demographic factors.

### Intervention dosage effects

On average, the intervention participants attended seven sessions (SD = 3) with the intervention “dose” ranging between 0 and 10 sessions (median = 8). Over 74 % (55/74) attended seven or more sessions while 36 % (27/74) attended all ten sessions. The intervention “dose” was not found to be statistically significantly associated with child BMI z-scores at post-intervention without or with adjustment for baseline sociodemographic characteristics (*p* = 0.15 and *p* = 0.21, respectively).

### Pubertal onset by treatment group effect

Post-hoc stratification to examine whether the study intervention had a differential effect on BMI z-scores by pubertal onset indicated a statistically significant pubertal onset-by-treatment group interaction in both post-intervention (*p* = 0.01) and follow-up models (*p* = 0.02). Subsequent sub-group analysis indicated a treatment group effect among prepubescent children only, at both post-intervention and follow-up time points (*p* = 0.03 and *p* = 0.001, respectively) in the longitudinal model. The adjusted mean BMI z-score difference between the control and intervention groups among prepubescent children at post-intervention was 0.18 (SE = 0.08; 95 % CI: 0.01, 0.34), corresponding to an estimated decrease in expected average weight gain of about 1.00 kg for a 9-year-old at the 75^th^ percentile for baseline BMI and of about 1.20 kg, if starting at the 85^th^ percentile for BMI. Likewise, for an 11-year-old, the expected decreases were more pronounced at about 1.40 kg and 1.70 kg, accordingly.

### Longitudinal effect of the intervention on family meal frequency

The majority of participants (60-70 %) reported “frequent” (5 or more) family dinners per week at all time points. No statistically significant group, time or group-by-time effects on the probability of having frequent family dinners were observed when post-intervention and follow-up time points were modeled while controlling for baseline outcome values and adjusting for baseline demographic and SES characteristics. Frequent family dinners at baseline was the strongest predictor of having frequent family meals later (*p*< 0.001). Economic assistance receipt at baseline was significantly associated with a lower probability of having frequent family dinners (*p* = 0.02). The predicted probability of having frequent family dinners for an intervention participant was higher (albeit not significant) than that for a control participant at post-intervention (0.72 and 0.56, respectively), whereas the probabilities were similar at follow-up (0.75 and 0.74, accordingly).

## Discussion

The present RCT reported on the efficacy of a family meals-focused intervention to prevent excess weight gain among 8-12-year-old children. The HOME Plus study was unique in that it was prevention- and family-focused with BMI as the primary outcome. Overall, the intervention effect on BMI z-scores did not reach statistical significance; however, modest and promising decreases in excess weight gain were observed at post-intervention and were sustained. Our post-hoc stratification analysis indicating significantly lower BMI z-scores for intervention children compared to their control group counterparts, among the prepubescent children only, is promising but should be viewed cautiously; further examination is warranted.

The present study exploratory findings suggest family-meal focused programming may potentially be efficacious to prevent excess weight gain among children prior to pubertal onset, as our cut-point for stratification was on the low end of development and consistent with prepubertal definitions[31]. Our findings support the conclusions of several reviews showing that associations between family meal frequency and weight status/BMI are robust in younger children [9, 21]. Perhaps the persistence of BMI across development is strong enough that intervention timing is critical and should occur early during development. Moreover, we were unable to tease apart the potential biological and psychosocial changes associated with maturation; we suspect that engagement in a family-focused intervention and changes in BMI z-scores may be influenced by both. It is unlikely our main effect not reaching statistical significance was due to lack of intervention fidelity or family engagement given the high fidelity and attendance rates. Rigorous family meals research with younger populations is needed, particularly to examine the effects of psychosocial changes during this age, related family dynamics, and possible interactions with biological influences.

Our study programming attempted to target children comprising a wide range of ages and maturational stages. Although we believe the intervention program was age-appropriate for 8-12-year-olds, some participants were pubescent and perhaps, given the strength of biological influences at this time, additional content and/or strategies are needed to have a larger impact on BMI. Additionally, reviews have consistently indicated longer-term programs (12+ months) are more effective than shorter-term programs (<12 months) [3, 8, 46]. HOME Plus was a 10-month program that may be shy of the length of time necessary for substantial BMI change. However, based on HOME Plus intervention-participant feedback, 88 % indicated a longer community-based program would not be preferred. Increased costs would also prohibit programs of much longer length given the current funding climate. Alternatively, our inability to show a planned difference in our primary outcome may be due to an unmeasured influential variable that we failed to address or consider.

The present study had several strengths. The full scale study’s design and intervention program had been pilot tested with similarly successful recruitment, retention and acceptability[23]. The theoretical framework, study design, methodology and measurement were high quality and the intervention had high fidelity. Intervention efficacy was tested using a RCT with both post-intervention and long-term follow-up; many studies do not test for sustainability [9]. The main outcome measurement was strong as it was measured objectively by trained staff. The randomization procedure was effective, with no baseline treatment group imbalance. The combination of delivering the intervention using both group- and individualized-approaches provided multiple pathways for behavior change and the use of standardized manuals and continual monitoring of intervention delivery demonstrated intervention integrity. An additional strength of the intervention was the delivery in real-world community settings, demonstrating successful partnerships with community organizations. Providing convenient intervention and measurement locations and facilitating participation by offering transportation, childcare and flexible scheduling were key to successful engagement and high retention rates.

Several limitations should be noted. The generalizability of study findings is limited as participants, who self-selected into the study, may have done so because of interests in cooking and family meals, and thus may have been more receptive to behavioral change. However, *all* of our families identified important behavioral goals for improvement since we provided a menu of goals and allowed flexibility in goal selection. Also, families were recruited across a large spectrum of income levels and racial diversity given the area population and appeared to be representative. In addition, our inclusion criteria of BMI ≥ 50^th^ percentile may have affected findings. Including a large proportion of normal weight children may have made it more difficult for us to see programmatic effects on BMI; as expected and shown in the description above, the intervention effect was more pronounced for heavier children. We were unable to model incidence of overweight/obesity by treatment group due to an insufficient number of children in our sample who were at normal weight at baseline but considered overweight/obese at post-intervention. Finally, at baseline, 61 % of families participating in HOME Plus were eating family dinner together 5 or more times per week, limiting the program’s ability to increase family meal frequency which could have also diminished the effect on weight-related outcomes. Our high participation rates in the intervention sessions meant that we did not observe a dose–response intervention effect on BMI z-score. Future researchers in this area may consider recruiting only families that report infrequent family meals to potentially have a larger preventative effect on excess weight gain.

Future research is needed in several areas of childhood obesity prevention study design and intervention development and delivery. Prevention of excess weight gain among normal weight youth may be more difficult than targeting weight reductions in overweight or obese youth [14] and may take longer to show [46]. In relation to the development and implementation of interventions, perhaps tailoring for within-group differences is needed [47] by pubertal development. Given that the present study was conducted with urban families, many of whom were eating frequent family meals, future family meals-focused interventions should prioritize engaging families in rural communities and including families who do not already frequently eat together.

## Conclusions

The HOME Plus study was a high quality study that tested a family meals-focused intervention with parents and children for the prevention of excess weight gain. Although we did not have a statistically significant overall intervention effect in our primary analysis, we observed modest but promising reductions in excess weight gain across age and weight status and significant effect modification by pubertal onset, suggesting potential weight-related effects for family meals-focused interventions among prepubescent children. Further research in this area is warranted.

## Abbreviations

BMI: body mass index
CDC: Center for Disease Control
HOME: Healthy Home Offerings via the Mealtime Environment
IRB: Institutional Review Board
RCT: randomized controlled trial
US: United States
